# Epithelial stem cell culture: modeling human disease and applications for regenerative medicine

**DOI:** 10.1186/s41232-017-0034-9

**Published:** 2017-02-06

**Authors:** Yusuke Yamamoto, Takahiro Ochiya

**Affiliations:** Division of Molecular and Cellular Medicine, National Cancer Center Research Institute, 5-1-1, Tsukiji, Chuo-ku, Tokyo, 104-0045 Japan

**Keywords:** Epithelial stem cells, Feeder cells, Small molecules, 3D culture

## Abstract

The inability to maintain the immaturity of stem cell populations in vitro restricts the long-term expansion of various types of human epithelial stem cells. However, recent technical advances in epithelial stem cell culture have led to the development of novel in vitro strategies for regenerating epithelial tissues and for closely mimicking human diseases such as cancer and inflammation. Specifically, improvements in culture conditions provided by small molecules in combination with three-dimensional (3D) culture approaches have facilitated the establishment of in vitro systems that recapitulate biological properties in epithelial organs, and these systems may be used to model disease. In this review article, we describe the biological significance of technical improvements in the development of these methods, focusing on human epithelial cells, including stratified and columnar epithelial cells. We also discuss the potential and future perspectives of this technology, which is only beginning to be explored.

## Background

The isolation and long-term expansion of primary cells, particularly stem/progenitor populations, are fundamental and important basic techniques in various biological fields, including developmental biology and stem cell biology, and medical science. Cells in stratified and columnar epithelial tissues are highly regenerative and disproportionately accountable for many human cancers; however, cloning adult stem cells is limited by difficulties in maintaining these cells in an immature state. In recent years, technical innovations have resulted in rapid and dramatic progress in stem cell biology, such as the use of small molecules and growth factors to mimic tissue niche environments and facilitating “Organoid culture” [1].

In 1975, Rheinwald and Green established the first successful example of human adult stem cell culture using human keratinocytes [2]. Specifically, they maintained human keratinocytes long-term in combination with a sublethally irradiated mouse fibroblast cell line, 3T3-J2. Although they did not use the term “stem cells” for cloned keratinocytes grown on 3T3 cells, Green and colleagues found colonies with the remarkable capacity to divide and form new colonies after passage, which they termed “Holoclones” [3]. These holoclones consists of small, immature cells that all exhibited intense nuclear staining with p63, a master regulator of stemness, in stratified epithelial cells [4]. In the stratified epithelium, including skin, lung bronchia, mammary gland, and bladder urothelium, the stem cell population was mainly localized in the basal layer, and immature cells were stained with p63, consistent with the in vitro studies [5]. Significantly, isolated and expanded human keratinocytes from autologous skin have been successfully grafted to burn patients and regenerated a permanent epidermis resembling that result from split-thickness skin grafts [6, 7]. Notably, the same procedure has been applied to isolate and expand human corneal epithelial cells for transplantation [8–10]. Although this technology was limited to stem cells in the epidermis and cornea at that time, Green and colleagues created the foundation for cloning human adult stem cells in the fields of basic biology and regenerative medicine.

In this review article, we provide an overview of recent research progress and accumulating evidence of a cell culture system that has led to technical breakthroughs in epithelial cell technologies. Novel culture strategies for both stratified epithelial cells and columnar epithelial cells have enabled human epithelial development to be recapitulated and can be used to generate a human disease model in vitro. We also discuss the potential and possible applications of normal epithelial cell culture technologies for regenerative medicine and highlight a cancer cell culture system that reproduces individual patient phenotypes.

### Stratified epithelial cell culture

**Fig. 1:**
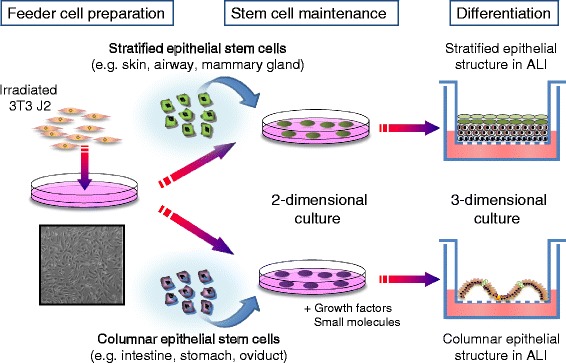
Schematic of the cell culture process for human stratified and columnar epithelial stem cells on a 3T3 mouse feeder layer. For stratified epithelial stem cells, they are isolated from biopsy or surgical specimens are plated on a 3T3 layer for a long-term culture. For columnar epithelial stem cells, they are plated on a 3T3 layer with defined factors which are essential for stem cell growth and maintenance. Morphologically immature colonies (packed colonies with small cells) of epithelial stem cells are mechanically picked-up for further homogeneous expansion. In the ALI culture, the cells undergo differentiation into mature cell types in a Transwell

In stratified epithelial tissues, including glandular and pseudostratified epithelium, p63+ cells, which are localized on the basement membrane, can self-renew to maintain stem/progenitor populations and give rise to progeny that form functional tissues [4, 5]. As mentioned above, the cloning and expansion of epithelial stem cells, such as skin keratinocytes and corneal epithelial cells, have been well-established in co-culture systems with irradiated mouse 3T3-J2 fibroblasts. However, this standard protocol has largely been limited to the long-term culture of keratinocytes and corneal cells. Nevertheless, cloned stem cells from thymic epithelia have been reported, as has the isolation of thymic epithelial stem cells from diverse species, including human cells, cultured with a 3T3 feeder system [4, 11, 12]. Furthermore, Frey and colleagues recently applied the 3T3 feeder method to isolate urothelial stem cells that expressed sonic hedgehog and resided in the basal layer of the bladder urothelium [13]. These urothelial stem cells from isolated human and porcine tissue were stably grown on a 3T3 feeder layer and were able to give rise to multiple cell lineages, including p63+ basal cells and Uroplakin 2+ and 3+ urothelial cells, after renal capsule transplantation in nude mice. In 2011, Pooja et al. exploited the 3T3 culture system to isolate three types of human airway epithelial stem cells, i.e., nasal, tracheal and distal airway stem cells, and found that these airway epithelial stem cells exhibited distinct cellular phenotypes after in vitro differentiation, although the immature stem cell clones appeared to be morphologically indistinguishable (Fig. 1) [14]. In a follow-up study, the transplantation of mouse tracheal and distal airway epithelial stem cells demonstrated that distal airway stem cells were readily incorporated into H1N1 influenza-damaged lung tissue and differentiated into multiple epithelial cell types, i.e., bronchioles and alveoli, whereas transplanted tracheal stem cells were localized only in major airways [15]. Clonogenic stem cells were also isolated from human esophagus endoscopic biopsy samples, and these cells were able to form well differentiated, stratified squamous epithelia-like structures in an air liquid interface (ALI) culture system [16].

Schlegel and colleagues reported that a Rho-associated protein kinase (ROCK) inhibitor in combination with 3T3 feeder cells significantly increased the proliferative capacity of epithelial stem cells, including human keratinocytes, prostate cells, and mammary gland cells, and they termed this phenomenon “conditional reprogramming” [17, 18]. The ability to efficiently generate epithelial stem cell cultures from patients provides critical and valuable insights into cell-based diagnostics and therapeutics [19]. More recently, Rajagopal and colleagues showed that the TGFβ/BMP/SMAD signaling pathway is important in various epithelial tissues, including ectoderm-derived skin and mammary gland tissue, endoderm-derived esophagus and prostate tissue, and mesoderm-derived epididymis. They discovered that the dual inhibition of SMAD signaling (the BMP signal was blocked by DMH-1 and the TGFβ signal was inhibited by A-83-01) facilitated the stable propagation of human and mouse epithelial basal cell populations. Surprisingly, dual TGFβ/BMP inhibition enabled the robust expansion of epithelial stem cells without the need for mouse 3T3 feeder cells.

Collectively, these technical advances, in combination with small molecules and feeder cells, can be used to continuously and efficiently expand stratified epithelial stem/progenitor populations in vitro. Another breakthrough in stratified epithelial culture, organoid culture, has been utilized to expand both basal and luminal human prostate progenitors. These human luminal progenitors were multipotent and formed prostate gland-like structures in vitro [20]. However, generating three-dimensional structures consisting of stratified or pseudostratified epithelia to recapitulate authentic in vivo architecture remains challenging, although many researchers have reported spheroid and organoid cultures. This problem may be solved by establishing a method to facilitate self-organization, as performed in pluripotent stem cell-derived tissues [21, 22].

### Columnar epithelial cell culture

Although intestinal stem cells possess the remarkable capacity to proliferate at a high turnover rate to maintain intestinal epithelia, and hepatocytes are highly regenerative in response to damage, the ability to clone stem cell populations from columnar epithelial cells is severely limited, presumably due to a lack of tissue niche signals in vitro. Over the past decade, Clevers and colleagues discovered LGR5 (leucine-rich repeat-containing G-protein coupled receptor 5), an intestinal stem cell marker, in a sophisticated mouse model (Lgr5-EGFP-ires-CreERT2 mice crossed with the Cre-activated Rosa26 LacZ reporter) and established a mouse intestinal organoid culture method that consists of villus-like structures and crypt-like zones with multiple intestinal cell types [23]. In combination with growth factors and small-molecule cocktails, an isolated LGR5+ stem cell fraction was suspended in Matrigel and cultured long-term [24]. Modifying the culture condition with the use of nicotinamide, a p38 and TGFβ receptor inhibitor, human epithelial cells isolated from the small intestine and colon were able to infinitely expand long-term in vitro [25, 26]. This technique is applicable to culture other types of cells, such as pancreatic duct cells [27] and hepatocytes [28], and facilitated revolutionary advances in columnar epithelial cell culture.

Organoid culture employs a Matrigel-based 3D culture platform and can be extensively used to stably culture diverse types of adult epithelial cells, including stratified epithelial cells, with stem/progenitor cell populations [1]. However, the ability to rapidly and efficiently propagate a fraction of uniform stem cells in vitro is also useful and important for the detailed study of self-renewal and fate specification in tissue stem cells and possible future applications of cell transplantation for regenerative medicine. Xian and colleagues recently developed a novel culture system for the homogeneous expansion of human fetal intestinal stem cells, including small intestine and colon cells. This system employed a 3T3 mouse feeder layer in combination with growth factors and signal pathway inhibitors to robustly expand human columnar epithelial stem cells (Fig. 1) [29, 30]. Moreover, more than 50% of intestinal stem cells grown on 3T3 fibroblasts were able to form colonies. In the mammalian intestine, defined niche factors, such as Wnt and Notch signals, are essential for governing the stemness of intestinal stem cells at the crypt base. Furthermore, Paneth cells, which are also located at the crypt base, arise from stem cells and act as the stem cell niche by providing essential factors in a paracrine manner. Because organoid cultures consist of stem cells and various derivatives, such as Paneth cells, niche factors are autonomously supplied [31]. By contrast, because a pure population of intestinal stem cells is grown on a 3T3 feeder layer, the cells cannot secrete niche factors. Therefore, extrinsic factors resembling niche factors need to be supplemented. In addition to the stem cell maintenance protocol, a differentiation protocol has been established in an ALI culture model to give rise to at least four types of major intestinal cells, i.e., Paneth cells, entero-endocrine cells, goblet cells, and enterocytes (intestinal absorptive cells) [29]. The formation of intestinal villus-like structures was observed according to the original tissue types, such small intestine and colon tissues (Fig. 1). In a different ALI culture approach, Kuo and colleagues robustly cultured small pieces of mouse neonatal intestine with a stromal element long-term [32].

The same strategy was also applied to clone human gastric stem cells obtained from endoscopic biopsy. Specifically, clonogenic gastric cells were stably expanded on a 3T3 feeder layer in combination with growth factors and small molecules and differentiated into gastric epithelial lineages typically found in the stomach, such as pepsinogen-expressing chief cells [16]. In addition to cloned digestive organ stem cells, oviduct progenitor cells from the distal uterus tube were also able to infinitely propagate on a 3T3 feeder layer in the presence of niche factors [33]. The distal oviduct, fimbria epithelium, is a simple columnar epithelium layer that consists of the following two types of cells: ciliated cells, which enhance the transport of gametes, and secretory cells, which secrete mucus. Using a slight modification of the differentiation protocol for intestinal stem cells, long-term ALI-cultured oviductal stem cells gave rise to a 3D architecture, containing both ciliated and secretory cells, that was reminiscent of the in vivo epithelium structure [34]. The ability to produce epithelial lineages with proper cell types from a stem cell population could be a useful tool to study physiological epithelial development and homeostasis and develop acute and chronic disease models in vitro.

### Cancer cell culture

Since the first cancer cell line, the HeLa cell line, was established from a cervical cancer patient in 1951 [35], cancer cell lines established from a wide variety of cancer types have been widely used to study the pathobiology of cancer and provided opportunities to generate in vivo xenograft models and test anti-cancer drugs in vitro and in vivo. Although tremendous advances have been made in cancer biology using cancer cell lines, the results obtained using these cells may not sufficiently reflect the complexity of the disease as originally expected because cancer exhibits interpatient and intratumor heterogeneity, as revealed by recent advances in next-generation sequencing [36]. To more precisely reflect cancer phenotypes, including the patient’s gene mutation status and pathology, Welm and colleagues developed patient-derived xenograft (PDX) models of breast cancer in nonobese diabetic severe combined immunodeficiency (NOD-SCID) mice that maintained the essential features of the original tumors and exhibited metastatic capacity to specific sites [37]. In addition to the breast cancer model, the establishment of various types of solid tumors demonstrated the feasibility of PDX models [38], which are anticipated to accelerate the preclinical testing of new cancer therapies and help realize the goal of “personalized medicine”.

Culture methods for adult stem cells, such as organoid and feeder systems, are also applicable to different approaches that use patient-derived cancer cells. Specifically, Clevers and colleagues reported that organoid culture can be used to model pancreas [39], prostate [40], and colorectal cancer [41] and showed that the original cancer traits, including genetic heterogeneity and drug sensitivity, can be recapitulated. Therefore, they termed this system a “living organoid biobank”. These technologies could also be used to isolate a stem cell population from a precancerous lesion, such as Barrett’s esophagus, a precursor of human esophageal adenocarcinoma [16, 25]. Isolated and expanded Barrett’s esophagus stem cells were transformed by introducing SV40 large T antigen, hTERT, and c-myc and xenografted into immunocompromised NSG (NOD.Cg-Prkdcscid Il2rgtm1Wjl/SzJ) mice [16]. As expected, Barrett’s esophagus stem cells transformed into esophageal adenocarcinoma-like tumors in mice. A similar approach demonstrated that human oviductal stem cells were the cell of origin in high-grade serous ovarian epithelial cancer [34]. This finding corroborates recent human pathology and the transgenic mouse model evidence, which indicated that the distal oviductal epithelium is the tissue of origin for this cancer [42, 43]. In combination with the CRISPR/Cas9 system, normal colon stem cells were sequentially transformed by introducing the driver mutations that are frequently detected in colorectal cancer [44, 45]. The resultant cells were allowed to form xenografts in the kidney capsule and exhibited progressive transformation into adenocarcinoma-like phenotypes characterized by invasive and metastatic properties. Overall, the ability to isolate and culture cells from tumor- and patient-matched normal epithelial tissues facilitates the production of a platform that not only complements classic in vivo animal work in the field of cancer biology but also facilitates patient-specific genetics and genomics approaches in vitro.

### Modeling inflammation disease with adult stem cells

Modeling human disease is hindered by the limited accessibility of human diseased tissues. Nevertheless, advances in adult stem cell cultivation have allowed us to reproduce disease phenotypes in vitro by expanding stem cells and deriving mature cell types from small human biopsy samples. Because 3D culture methods, such as ALI and organoid culture, provide structures that consist of multiple cell types and resemble the epithelium architecture observed in vivo, they should be suitable for studying inflammatory diseases, including infectious and hereditary diseases. Specifically, reproducing the disease phenotype is simple when the pathogen (or main cause) and targeted cell type are known.

Pseudomembranous colitis (PMC) is caused by a disproportionally increased population of Clostridium difficile (C. difficile) after antibiotics treatment. C. difficile is a Gram-positive, spore-forming bacterium, and produces the high-molecular-weight toxins TcdA and TcdB, which induce fluid secretion, inflammation, and colonic tissue damage. Colonic epithelial cells differentiated from clonogenic colonic stem cells in ALI culture were challenged with these toxins, which caused devastating epithelial damage in a time- and dose-dependent manner. This result indicated that the 3D culture model may be used to represent C. difficile pathology [29]. Similarly, the effect of Helicobacter pylori (H. pylori) infection, which causes chronic gastritis, gastric ulcers and cancer, was studied by microinjecting H. pylori into organoid cultures. Bacteria-infected organoid cultures exhibited increased inflammation, such as NF-kB activation and IL8 induction, and IL8 expression was significantly higher in gland-type organoid cultures than in pit-type organoid cultures [46].

Adult stem cells have also been used to model hereditary disease. Beekman and colleagues reported an intestinal organoid culture derived from cystic fibrosis (CF) patients. CF is caused by mutations in the cystic fibrosis transmembrane conductance regulator (CFTR), which is normally expressed in the epithelial cells of many organs, such as lung and digestive tissues. Although normal intestinal organoid cultures exhibited robust swelling in response to Forskolin, the swelling response was not observed in CF organoid cultures [47]. Moreover, when the mutated CFTR locus was corrected using the CRISPR/Cas9 technology in intestinal organoids of CF patients, the corrected genes were shown to functionally work [48]. Therefore, in vitro differentiation of adult stem cells, resembling in vivo phenotypes with multiple cell types in combination with gene editing technologies, provides powerful means for treating human disease and may provide direct insight into human pathology.

### Application of epithelial stem cells for regenerative medicine

Despite promising strategies that use human embryonic stem (ES) cells and induced pluripotent stem (iPS) cells for applications in regenerative medicine, few clinical trials of these strategies are ongoing, which is in part due to difficulties in lineage specification and the possibility of tumorigenesis. Because adult stem cells are essentially committed to specific tissue types, producing intended cell types is relatively easy, and the potential risk for tumorigenesis is low. Thus, therapeutic approaches are aiming to use adult stem cells as the cell source for transplantation. Although Green and colleagues established the human keratinocyte culture method in 1975 and the cultured cells were transplantable into patients with burns or chemical injuries, the long-term cultivation of other types of adult stem cells was subject to significant technical barriers. As described above, recent technical advances overcame this limitation for diverse types of epithelial cells. Hence, the ability to rapidly and efficiently expand stem cell populations is valuable for their use in regenerative medicine.

For example, mouse Lgr5+ colonic stem cells have been expanded in organoid culture and transplanted into the damaged mouse colon, and engrafted cells that were able to self-renew and differentiated were detected even after 25 weeks [49]. In a different approach, Zhang K and colleagues harnessed engineered adult stem cells for a transplantation study. First, they successfully cultured corneal epithelial cells in a dish without feeder cells and then found that Pax6 is a key transcription factor that differentiates corneal stem cells (CSCs) from skin keratinocytes. Surprisingly, Pax6-overexpression in keratinocytes induced limbal stem cell-like cells, and these cells could be transplanted into the injured corneas of rabbits [50]. Because keratinocytes are more readily accessible than CSCs, this method may be applicable for the treatment of human eye disease. More recently, Liu et al. reported an attractive approach for tissue repair and regeneration that used endogenous stem cells. In their study, lens epithelial stem cells (LECs) that expressed Pax6 and Bmi1 were characterized and exhibited regenerative potential in vivo. A surgical cataract removal method that preserves endogenous LECs was employed, and these LECs contributed to the spontaneous regeneration of lenses with visual function in rabbits, macaques, and human infants. This method could be a therapeutic breakthrough for cataract treatment and potentially replace artificial intraocular lens implantation [51].

Because of the high turnover rates of many epithelial cells, transplanting stem cell populations is essential for long-term tissue maintenance. Theoretically, a single stem cell can reconstitute whole tissues, and several research groups empirically demonstrated this notion [52, 53]. Despite the potential of pluripotent stem cells (PSCs), which can give rise to all cell types, PSC-derived tissue stem cells likely cannot be maintained in the immature state in vitro. Therefore, the use of adult stem cells for regenerative medicine presents a significant advantage.

## Conclusions

In recent years, remarkable progress has been made in the development of in vitro culture system for epithelial stem cells. The realization of the long-term culture of epithelial stem cells allows us not only to reproduce physiological events in vitro but also enables the development of therapeutic platforms based on cell transplantation. An increasing number of studies of epithelial stem cells clearly indicated that understanding the basic biology of these cells will be closely linked with clinical studies of human disease pathology, such as cancer and inflammation. The interactions of biological networks during tissue development and disease progression are complex at the cellular and molecular levels. Building an in vitro epithelial structure model can simplify this complexity and provide comprehensive views of epithelial physiology and pathophysiology. Moreover, in vitro epithelial models can easily be combined with genomic and epigenetic approaches and single-cell analyses. In addition, genome editing, e.g., the CRISPR-Cas9 system, can also be readily incorporated into the model. One drawback of in vitro epithelial structure models derived from the stem cells is that epithelial structures lack stromal populations. Although a simplified system provides direct insight into epithelial physiology in most cases, the interaction between different cell types is important for reproducing a genuine phenotype because all tissues consist of multiple cell types, such as epithelial cells, endothelial cells, mesothelial cells, fibroblasts, and hematopoietic cells. One possible solution to this problem is a self-organizing method, in which several cell types are mixed in vitro and spontaneously form actual organ-like structures. Although improvements are required to recapitulate the in vivo behavior of human organs, the ability to expand epithelial stem cells and generate a 3D structure model holds great promise for both basic and clinical research.

## Abbreviations

3D: Three-dimentional
ALI: Air-liquid interface
C. difficile: Clostridium difficile
CF: Cystic fibrosis
CFTR: Cystic fibrosis transmembrane conductance regulator
CSC: Corneal stem cell
ES: Embryonic stem
H. pylori: Helicobacter pylori
iPS: Induced pluripotent stem
LEC: Lens epithelial stem cell
LGR5: Leucine-rich repeat-containing G-protein coupled receptor 5
NOD-SCID: Nonobese diabetic severe combined immunodeficiency
PDX: Patient-derived xenograft
PMC: Pseudomembranous colitis
PSC: Pluripotent stem cell
ROCK: Rho-associated protein kinase
